# Two to tango: DTT1 regulates barley tapetum transition as part of a paired key

**DOI:** 10.1093/plcell/koaf249

**Published:** 2025-10-16

**Authors:** Julie Robinson

**Affiliations:** Assistant Features Editor, The Plant Cell, American Society of Plant Biologists; HudsonAlpha Institute for Biotechnology, Huntsville, AL 35806, USA

As with the formation of all organs, anther development requires the precise coordination of multiple components to ensure faithful cell differentiation and the formation of a functional organ. In the case of reproductive organs, this is of the utmost importance for the survival of the organism on an evolutionary scale.

The sequential morphological changes that comprise anther development have been well characterized in model species; however, many questions still remain regarding the molecular pathways behind these changes and how well conserved they are among different species, particularly between monocots and dicots. **In new work, Miaoyuan Hua and colleagues** (Hua et al. 2025) identify DEFECTIVE TAPETUM TRANSITION1 (DTT1) as a protein with a key role in anther development in barley and characterize its function. DTT1 is an ortholog of TDR-INTERACTING PROTEIN2 (TIP2), a basic helix-loop-helix (bHLH) protein in rice that has been well characterized for its important role in anther development (Fu et al. 2014).

First, Hua and colleagues examined the expression pattern of *DTT1* in barley and determined that it may be involved in anther development, just like its rice ortholog. Mutating *dtt1* indeed resulted in male sterility, cementing its role as a key player in anther formation. Taking a closer look at what caused this sterility, the researchers found that mutant anthers failed to undergo the transition from stage 6 to stage 7 of the 14-stage process. During this transition, the tapetum, a cell layer that provides nutrients to developing pollen mother cells, usually undergoes differentiation that allows it to go on to regulate meiosis within the pollen mother cells. If differentiation fails at this stage, then the pollen does not develop properly and anther development becomes arrested, leading to male sterility.

To better understand the mechanism by which a lack of DTT1 results in failure to transition from stage 6 to stage 7 of anther development, the researchers performed differential gene expression analysis of RNA-sequencing data to identify potential targets of DTT1. Since the MYB transcription factor TDF1 is known to act downstream of DTT1, they eliminated genes that also show differential expression upon *tdf1* mutation. The resulting 225 genes showed enrichment for tapetum development and metabolic processes. Among the identified genes were the bHLH transcription factors AMS and EAT1 as well as other MYB transcription factors.

bHLH proteins typically bind DNA as homodimers (two of the same protein) or heterodimers (two different proteins) via their basic regions (Chapman-Smith and Whitelaw 2006). Hua and colleagues investigated whether DTT1 forms homodimers, heterodimers, or both by performing protein interaction assays. They found that DTT1 did not homodimerize when expressed under the AtUbi10 promoter, but did heterodimerize with the bHLH transcription factor DYSFUNCTIONAL TAPETUM1 (DYT1), which is known to be a key player in anther development. A yeast two-hybrid assay showed that formation of this heterodimer was mediated by a highly conserved IKL motif within the ACT-like/bHLH protein interaction and function (BIF) domain of DTT1. However, knowing that most bHLH proteins form homodimers and that DTT1 is a putative paralog of other bHLH proteins that do so, Hua and colleagues revisited their assays with the CaMV35S promoter, which overexpresses genes at a higher level than AtUbi10. Under CaMV35S overexpression, the researchers detected DTT1 homodimers. It must consequently be noted that, when overexpressing proteins for interaction assays, one should consider the stoichiometry of these proteins under physiological conditions. Protein truncation assays showed that DTT1–DYT1 heterodimerization occurs through interaction between their bHLH domains and their ACT-like/BIF domains with themselves and with each other, suggesting a role for the ACT-like/BIF domain in binding partner selection.

In mammals, E-box–like motifs serve as binding sites for transcription factors that regulate gene expression under hypoxic conditions (Kimura et al. 2001). In this work, Hua and colleagues show that the DTT1–DYT1 heterodimer binds both canonical E-box motifs and E-box–like motifs. The authors hypothesize that the DTT1–DYT1 complex may operate under hypoxic conditions, such as those found deep within the tapetum, noting that induction of hypoxia in maize has been shown to stimulate germ cell differentiation in germline-defective mutants (Kelliher and Walbot 2012). Altogether, Hua and colleagues show in this work that the DTT1–DYT1 heterodimer functions as a paired key to regulate the transition from stage 6 to stage 7 of anther development, during which this complex directly targets several key transcription factors. The identification and characterization of DTT1 in barley gives us more information about the evolution of anther development in monocots (Fig.).

**Figure.**
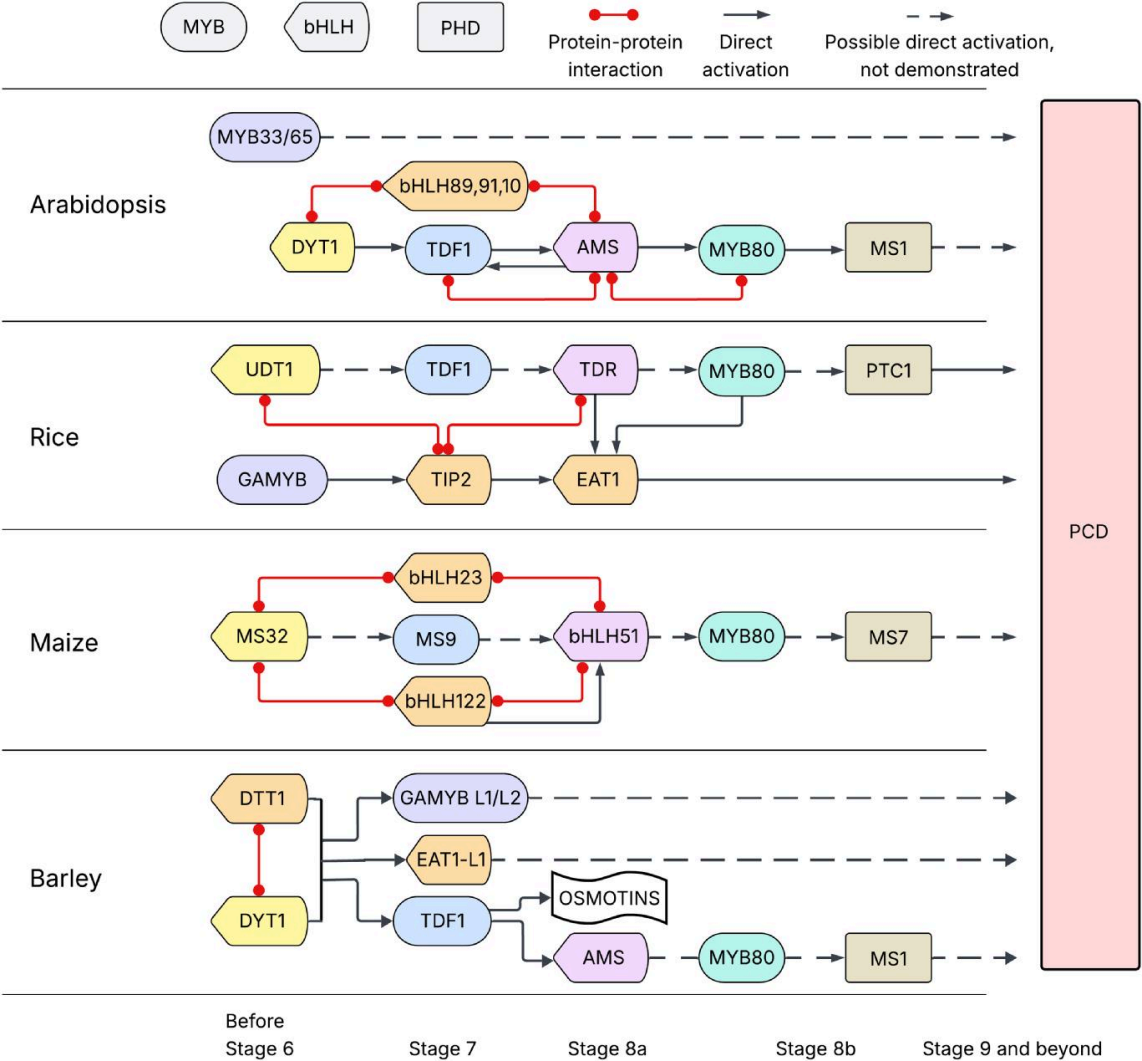
Anther development regulatory pathways in Arabidopsis, rice, maize, and barley with box colors linking orthologs. PCD, programmed cell death. Reprinted from Figure 9 of Hua et al. (2025).

## Recent related articles in *The Plant Cell*

- Tao et al. (2024) identified a regulatory cascade for starch biosynthesis during pollen development in rice.
- Chen et al. (2024) characterized an epigenetic mechanism involving polycomb repressive complexes by which stamen development is regulated in Arabidopsis.
- Chen et al. (2023) identified and characterized a protein that regulates homeostasis of uridine diphosphate sugars in rice in the context of pollen wall development.

## Data Availability

No new data were generated or analyzed in support of this article.

## Dive Curated Terms

The following phenotypic, genotypic, and functional terms are of significance to the work described in this paper:

anther AmiGo: PO:0009066

tdf1 Gramene: AT3G28470
tdf1 Araport: AT3G28470

ams Gramene: AT2G16910
ams Araport: AT2G16910

tapetum AmiGo: PO:0025313
